# Evaluation of macrophage migration inhibitory factor as an imaging marker for hepatocellular carcinoma in murine models

**DOI:** 10.3109/00365521.2011.568517

**Published:** 2011-05-11

**Authors:** Chao Zhang, Ting Liang, Jing Song, Shiqin Jiang, Lili Qu, Guihua Hou

**Affiliations:** Key Laboratory for Experimental Teratology of the Ministry of Education and Institute of Experimental Nuclear Medicine, Shandong University School of Medicine, Jinan, Shandong, P.R. China

**Keywords:** Anti-MIF McAb, immunohistochemistry, MIF, radioiodine, RT-PCR, tumor imaging

## Abstract

***Objective***. Macrophage migration inhibitory factor (MIF) is considered as an important mediator in the pathogenesis of neoplasia. The aim of the present study was to evaluate whether MIF could be used as a marker for hepatocellular carcinoma (HCC) detection. ***Material and methods***. Biodistribution and whole-body autoradiography studies of ^131^I-labeled anti-MIF monoclonal antibody (McAb) and ^131^I-labeled control IgG were performed. The HCC-bearing mice were injected with 3.7 MBq of each agent and killed at 24, 48, and 72 h postinjection (p.i.). The organs, blood, and HCC tissues were removed from model mice, weighed, and counted using a gamma-counter. The expression of MIF mRNA and protein within HCC tissues was confirmed by RT-PCR and immunohistochemistry. ***Results***. HCCs in model mice could be adequately visualized at 24 h p.i. The target-to-non-target (T/NT) ratios were 6.72 ± 1.09 (24 h), 9.85 ± 0.81 (48 h), and 12.31 ± 0.57 (72 h) for ^131^I-labeled anti-MIF McAb group, whereas in the control group of ^131^I-IgG, T/NT ratios were 4.65 ± 0.63 (24 h), 6.12 ± 0.60 (48 h), and 8.23 ± 0.35 (72 h) (*p* < 0.05). MIF mRNA expression was twofold higher in the HCC tissues than in the healthy liver tissues. MIF protein expression was much higher in the HCC tissues than in controls. ***Conclusions***. Our findings suggested that ^131^I-anti-MIF McAb could be rapidly and specifically localized in tumors. Thus, MIF could be used as a marker for HCC tumor detection.

## Introduction

Hepatocellular carcinoma (HCC) is one of the most frequently reported malignant tumors in contemporary medicine and is becoming increasingly significant in clinical research [1,2]. Detection of HCC at an earlier stage is very important for patient's prognosis. For decades, α-fetoprotein (AFP) has been used as a serum marker for HCC; however, it is insufficient for HCC screening. The ability of abdominal imaging to detect HCCs has improved dramatically over the last few decades. Although ultrasonography (US) and computed tomography (CT) usually accurately depict HCC [3], attempts to improve the methods of staging and follow-up for patients with known HCC have led to the evaluation of nuclear imaging systems in HCC [4]. Therefore, efforts are being made to identify alternative tracers for screening and staging HCCs in patients at risk.

Macrophage migration inhibitory factor (MIF) has been considered as the most interesting factor because of its diverse functions [5]. Originally described as a product of activated T cells that inhibits the migration of macrophages, MIF was subsequently found to be involved in the development of pathological changes associated with several acute and chronic inflammatory disease processes, such as acute respiratory distress syndrome [6], septic shock [7], rheumatoid arthritis [8], atherosclerosis [9], glomerulonephritis [10], and allograft rejection [11]. Recent studies have shown that MIF plays an important role in the tumor formation [12].

MIF has been shown to promote cell proliferation and tumor angiogenesis [13], and over-expression of MIF has been reported in various types of cancers, including prostate tumors, breast cancer, colon carcinomas, HCCs, and glioblastoma. In the current study, we labeled anti-MIF monoclonal antibody (McAb) with radioiodine Na^131^I in order to determine the role of MIF in HCC progression. In addition, we investigated its biodistribution and imaging characteristics *in vivo* using an HCC tumor-bearing model in mice. The results of our study indicated that MIF could be used as a supplementary biomarker for the detection of HCCs.

## Material and methods

### Radioiodination of anti-MIF McAb

All commercially available chemicals were of analytic grade and anti-MIF McAb (R&D Systems), and IgG (ZSGB-BIO) were of pharmaceutical grade. Anti-MIF McAb and control IgG were iodinated with Na^131^I (specific activity 37 MBq/mg, China Institute of Atomic Energy) using the Iodogen technique. Radioiodinated anti-MIF McAb and control IgG were separated from free iodine using size exclusion columns (Sephadex G-25, Pharmacia). The specific activity of radioiodinated anti-MIF McAb is 28.56 GBq/μmol, and that of radioiodinated IgG is 27.63 GBq/μmol. The radiochemical purity of radioiodinated anti-MIF McAb is >95% (as revealed by paper chromatography), and that of radioiodinated IgG is >95% (paper chromatography). The immunological activities of ^131^I-labeled anti-MIF McAb were confirmed by ELISA (enzyme-linked immunosorbent assay) (data not shown).

### Preparation of tumor-bearing animal model

Cell culture and all animal experiments were performed in accordance with the principles of the institutional, national, and international guidelines for humane use of animals for research. The HCC tumor models were established by subcutaneous injection of 5 × 10^6^ H22 tumor cells into the front flank of female severe combined immunodeficient (SCID) mice (20–25 g, 6–8 weeks, Animal Center of Shandong University). Thyroid uptake was blocked by the addition of potassium iodide to the drinking water (0.1%) starting from 1 day before the experiments. When the tumor volume reached 500–600 mm^3^ (4 weeks after inoculation), the tumor-bearing nude mice were used for biodistribution and gamma imaging studies.

### Biodistribution of ^131^I-anti-MIF McAb

The HCC tumor-bearing mice (24 mice/group) were injected with 3.7 MBq ^131^I-anti-MIF McAb or ^131^I-IgG in 0.2 ml of PBS via the tail vein to evaluate the distribution in major organs of mice. The mice were sacrificed at 24, 48, and 72 h post-injection (p.i.). Blood, heart, liver, spleen, kidney, brain, lung, intestine, muscle, bone, and tumor were harvested, weighed, and measured for radioactivity in a gamma-counter (1480 Wizard 3, PerkinElmer Life Sciences, Boston, MA, USA). Organ uptake was calculated as the percentage of injected dose per gram of tissue (%ID/g). Values were expressed as mean ± SD (*n* = 8/group).

### Whole-body autoradiography

For whole-body autoradiography imaging, the HCC tumor-bearing mice were anesthetized with an intra-peritoneal injection of sodium pentobarbital at a dose of 45 mg/kg. Each animal was administered with 3.7 MBq of the ^131^I-anti-MIF McAb or ^131^I-IgG in 0.2 ml of PBS (*n* = 4/group). Mice were placed on their back on a storage phosphor screen plate in subdued light. Each mouse was exposed to the plate for 25 min. When the exposure needed to be stopped, the plate was immediately covered with an opaque plastic sheet, transferred to a scanner, and scanned by using typhoon trio + (laser red, 633 nm; pixel size, 200 mm; phosphor mode: best sensitivity). Serial images were acquired at 24, 48, and 72 h p.i.

### RT-PCR

The expression of MIF mRNA was evaluated by RT-PCR. Total RNA was isolated from HCC tissues and healthy liver tissues using TRIzol reagent (Invitrogen Life Technologies, Carlsbad, CA, USA), and DNA contaminant was removed by treatment with DNase I (Invitrogen Life Technologies, Carlsbad, CA, USA) according to the manufacturer's instructions. Total RNA (5 μg) was reverse transcribed by using Revert Aid™ First Strand cDNA Synthesis Kits (Fermentas), cDNAs amplified with murine MIF specific primer using TaKaRa PCR Amplification Kit (TaKaRa). The primers were designed as follows: MIF (368 bp), sense primer, 5′-CCATGCC-TATGTTCATCGTG-3′; and antisense primer, 5′-GAACAGCGGTGCAGGTAAGTG-3′. Cycling conditions for amplification were as follows: 4 min denaturation step at 94°C, followed by 35 cycles of 30 s at 94°C, 1 min at 55°C, and 1 s at 72°C. PCR products were analyzed on 1.5% agarose gels and stained with ethidium bromide. All the experiments were performed in duplicate, and they were repeated at least three times. GAPDH mRNA expression was used as a loading control.

### Histological and immunohistochemical analyses

HCC tissues and healthy liver tissues were fixed in 10% PBS-buffered formalin, and paraffin sections were stained with H&E and examined by light microscopy to assess the histological changes. Immunohistochemical analysis was performed using a SP-9002 Histostain™-Plus kit (ZSGB-BIO) according to the manufacturer's protocol. The sections were microscopically examined and the positively stained fields were observed. Ten fields per each section were observed.

### Statistical analysis

The data were analyzed using SPSS 11.0 software. Statistical analysis was performed using the unpaired Student's *t*-test. Difference was considered statistically significant when *p* was <0.05, and two-sided. All data were expressed as the means ± standard deviation (SD).

## Result

### Accumulation in the HCC tissues

**Figure 1:**
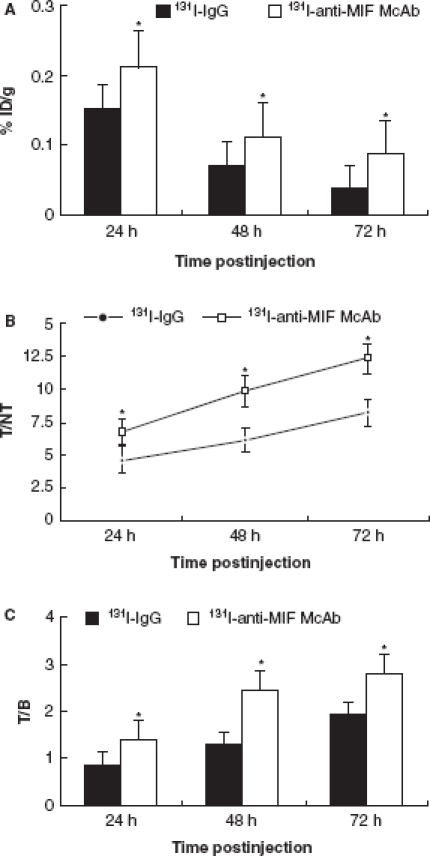
(A) Accumulation of ^131^I-anti-MIF McAb and ^131^I-IgG in the hepatocellular carcinoma (HCC) tissues (%ID/g). Values are means ± SD, *n* = 8 in all groups; *p < 0.05 vs. ^131^I-IgG. (B) Change of T/NT in the HCC tissues of ^131^I-anti-MIF and ^131^I-IgG McAb groups. Values are means ± SD, *n* = 8 in all groups; *p < 0.05 vs. ^131^I-IgG. (C) Change of T/B of ^131^I-anti-MIF McAb and ^131^I-IgG in HCC tissues. Values are means ± SD, *n* = 8 in all groups; *p < 0.05 vs. ^131^I-IgG.

The concentration of ^131^I-anti-MIF McAb or ^131^I-IgG in the HCC tissues was expressed as percentage of the initial dose (%ID/g). As shown in Figure 1A, both the radiopharmaceuticals significantly localized at sites of HCC tissues at 24 h p.i. The uptake of ^131^I-anti-MIF McAb in HCC tumors was 0.214 ± 0.021%ID/g at 24 h p.i. and retained to 0.086 ± 0.0013%ID/g at 72 h p.i. (*n* = 8/group). The uptake of ^131^I-anti-MIF McAb was significantly higher than that of ^131^I-IgG (*p* < 0.05). Target-to-non-target (T/NT) ratio for the ^131^I-anti-MIF McAb group was 6.72 at 24 h and increased continuously to 12.32 at 72 h. On the other hand, the T/NT ratio for the ^131^I-IgG group was 4.65 at 48 h and reached 8.23 at 72 h. ^131^I-anti-MIF McAb group showed the highest uptake (*p* < 0.05, see Figure 1B). Target-to-blood (T/B) ratios for the ^131^I-anti-MIF McAb group were 1.39 ± 0.21, 2.45 ± 0.13, and 2.79 ± 0.21 at 24, 48 and 72 h p.i., respectively. The T/B ratios for the ^131^I-anti-MIF McAb group were significantly higher than that of ^131^I-IgG group (*p* < 0.05, see Figure 1C).

Taken together, the data indicated that the ^131^I-labeled anti-MIF McAb was more specific and suitable for targeting HCC than the ^131^I-labeled IgG antibody.

### Imaging of HCC

**Figure 2:**
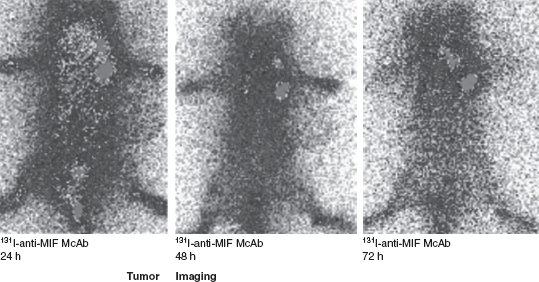
Images of the hepatocellular carcinoma (HCC) mice model. The ^131^I-anti-MIF McAb group had clear images in accordance with the high T/NT ratio.

The whole-body autoradiography images of the two radiotracers at 24, 48 and 72 h p.i. were shown in Figure 2. The HCC tumors of ^131^I-labeled anti-MIF McAb were clearly visible at 24 and 48 h p.i., with high contrast to the contralateral background. Comparative analysis of the scintigrams obtained at the three time points showed that the ^131^I-anti-MIF McAb group had remarkably clear images than those of the ^131^I-IgG group; this finding was in accordance with the high T/NT ratio (*p* < 0.05). These findings also indicate that the ^131^I-labeled anti-MIF McAb had higher specificity for targeting HCC than that of the ^131^I-labeled IgG antibody.

### MIF mRNA expression in HCC and healthy liver tissues

**Figure 3:**
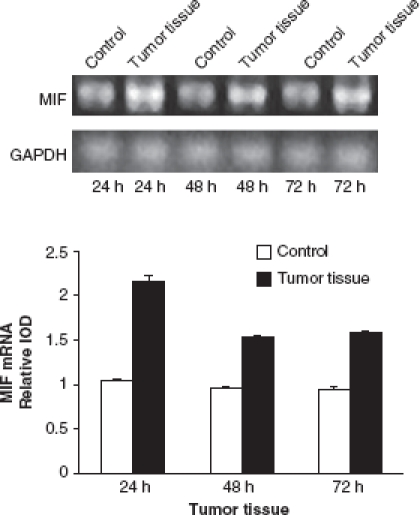
The expression of MIF mRNA in hepatocellular carcinoma (HCC) and healthy liver tissues. There were little changes in MIF gene expression in healthy liver tissues at the three time points. However, there was a twofold increase in MIF mRNA expression in HCC tissues at 24 h compared with healthy liver tissues (*p* < 0.05). The MIF mRNA levels in HCC tissues were keeping at a high level until 72 h compared with healthy liver tissues (*p* < 0.05). Semi-quantitative RT-PCR was performed in duplicate to minimize experimental error on the value calculated. All columnar values were expressed as means and standard deviations. A pattern of results was analyzed by repeating at least three times. *p* < 0.05 compared with the normal group.

To confirm that the high intake of ^131^I-labeled anti-MIF McAb in HCC tissues was due to the high expression of MIF in locus, we analyzed the expression of MIF in HCC tissues and healthy liver tissues. As shown in Figure 3, there were little changes in MIF gene expression pattern in healthy liver tissues at all the three time points. However, MIF mRNA expression in HCC tissues was twofold higher than that in healthy liver tissues at 24 h p.i. (*p* < 0.05). The MIF mRNA levels in HCC tissues remained elevated up to 72 h p.i., in contrast to expression in healthy liver tissues (*p* < 0.05). MIF was secreted from many kind of cells within tumor tissues, including activated lymphocytes, macrophages, and tumor cells; these cells may produce high levels of MIF as is noted in HCC cases.

### MIF protein expression

**Figure 4:**
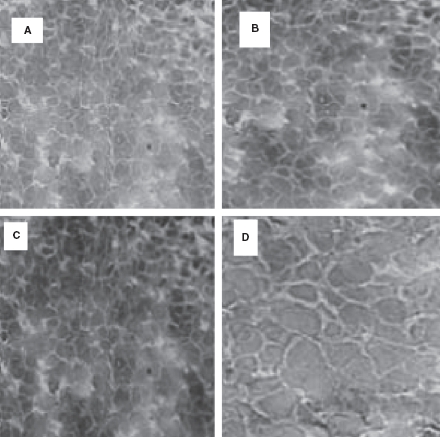
Expression of MIF *in vivo*. Expression of MIF protein was negative or weakly positive in healthy liver tissues (D). However, there was a significant increase in MIF expression in HCC specimens according to the time after injection in the first 48 h (A and B); and keeping the same level until 72 h (C).

Immunohistochemistry analysis showed that MIF was highly expressed in the HCC tissues (Figure 4). MIF expression significantly increased in the HCC specimens during the first 48 h p.i. (Figure 4A and B) and remained a high level until 72 h p.i. (Figure 4C). MIF protein expression was negative or weakly positive in healthy liver tissues (Figure 4D). We found a striking accumulation of macrophages in HCC tissues that may increase the expression of MIF. These results also showed that the high intake of ^131^I-labeled anti-MIF in locus was consistent with the high expression of MIF in HCC tissues.

## Discussion

HCC is the fifth most common cancer worldwide; its incidence is very high in China where it is the second most common cancer. Because many patients are at increased risk of HCC, there is a need to develop reliable, sensitive, and specific imaging markers that will aid the clinician in early diagnosis. Several diagnostic modalities such as ultrasound, CT, MRI have been used to image HCCs; however, there is no optimal modality to detect this disease at an early stage. Nuclear medicine has been reported to play a complementary role in the evaluation of questionable lesions using high tumor to non-tumor contrast imaging techniques [14]. Finding an ideal radiopharmaceutical for the rapid, accurate, and unequivocal identification of HCCs is necessary in nuclear medicine imaging. Over the past decades, research efforts have been directed toward the development of monoclonal antibodies as radiophar-maceuticals for application in fields of radioimmunoimaging [15].

In the present study, overexpression of MIF mRNA was found in HCC tissues compared with non-tumor tissues and correlated with the expression of MIF shown by immunohistology. Consistently, serial images of whole-body autoradiography showed that the ^131^I-anti-MIF McAb group had much more clear images compared with the control ^131^I-IgG group. MIF is a cytokine produced by T lymphocytes, macrophages, and tumor cells, and its expression is associated with cell proliferation, cell cycle entry, angiogenesis, and tumorigenesis, resulting in antigen-specific immune responses between ^131^I-anti-MIF McAb and MIF, while uptake of ^131^I-IgG control group in tumor tissues is caused by nonspecific binding to Fc fragments of normal IgG. Although radiolabeled human polyclonal IgG had received considerable attention as a radiopharmaceutical for nuclear medicine imaging [16], it is far from the ideal tumor imaging reagent because of its lack of specificity. There are several novel findings in this study. First, this is the first report using radioiodinated anti-MIF McAb to demonstrate that the positive MIF expression in HCC tissues correlates with a significantly high MIF mRNA expression. Second, radioiodinated anti-MIF McAb was used as a prospective HCC imaging agent and provided clear image in tumor radioimmunoimaging.

MIF was originally described as a lymphokine involved in delayed hypersensitivity and various macrophage functions, including production of proinflammatory cytokines, glucocorticoid-induced immunomodulator, as well as induction of metallo-proteinase. Furthermore, MIF is suggested to play a tumorigenic role because of its ability to drive activation responses by sustaining ERK1 and ERK2 MAP-kinase activation and by suppressing p53-dependent growth arrest and apoptosis; these functions suggest that MIF promotes malignant progression of tumors. Several studies have documented that MIF is expressed in the sera and liver of patients with HCC. Intracellular MIF mRNA and protein were found to be overexpressed in HCC. Patients with high MIF-expressing tumors had a worse disease-free survival [17]. These findings indicated that the HCC-progressive effect of MIF may be attributable to its action in inducing tumor-associated angiogenesis, immunomodulation, and alterations in the tumor suppressive pathway. The potential of an anti-MIF therapeutic strategy [18,19] has been highlighted by the ability of a neutralizing anti-MIF McAb to attenuate pro-inflammatory cytokine production. Therefore, MIF is considered as a potential target protein in many pathophysiological states.

Our study showed that the ^131^I-anti-MIF McAb has a high target-to-background ratio and has relatively low level of accumulation in non-target tissues, that its expression is highly associated with the local expression of MIF, and that it is considerably better than nonspecific IgG for detecting HCCs by radioimmunoimaging. Hence, we propose that MIF may be a good marker for early HCC detection.
